# Overall survival in patients with metastatic renal cell carcinoma in Russia, Kazakhstan, and Belarus: a report from the RENSUR3 registry

**DOI:** 10.1002/cnr2.1331

**Published:** 2020-12-25

**Authors:** Ilya Tsimafeyeu, Oxana Shatkovskaya, Sergei Krasny, Nurzhan Nurgaliev, Ilya Varlamov, Vladislav Petkau, Sufia Safina, Ruslan Zukov, Mikhail Mazhbich, Galina Statsenko, Sergey Varlamov, Olga Novikova, Igor Zaitsev, Pavel Moiseyev, Alexander Rolevich, Alesya Evmenenko, Irina Popova, Dilyara Kaidarova, Liubov Vladimirova

**Affiliations:** ^1^ Kidney Cancer Research Bureau Moscow Russia; ^2^ Institute of Oncology Hadassah Medical Moscow Moscow Russia; ^3^ Department of Strategic Development and International Relations Kazakh Institute of Oncology and Radiology Almaty Kazakhstan; ^4^ N.N. Alexandrov National Cancer Centre of Belarus Minsk Republic of Belarus; ^5^ Department of Urology Kazakh Institute of Oncology and Radiology Almaty Kazakhstan; ^6^ Department of Urology Altai Regional Cancer Center Barnaul Russia; ^7^ Out‐Patient Department Sverdlovsk Regional Oncological Dispensary Ekaterinburg Russia; ^8^ Chemotherapy Department Republican Clinical Oncology Dispensary Kazan Russia; ^9^ V.F. Voyno‐Yasenetsky Krasnoyarsk State Medical University Krasnoyarsk Russia; ^10^ Department of Urology Omsk Regional Cancer Center Omsk Russia; ^11^ Omsk Regional Cancer Center Omsk Russia; ^12^ Chemotherapy Department Khabarovsk Regional Cancer Center Khabarovsk Russia; ^13^ Department of Urology Astrakhan Regional Cancer Center Astrakhan Russia; ^14^ Organization of Anticancer Control N.N. Alexandrov National Cancer Centre of Belarus Minsk Republic of Belarus; ^15^ Laboratory of Oncourological Pathologies N.N. Alexandrov National Cancer Centre of Belarus Minsk Republic of Belarus; ^16^ Department of the Organization of Anticancer Control N.N. Alexandrov National Cancer Centre of Belarus Minsk Republic of Belarus; ^17^ Department of Medical Oncology National Medical Research Centre for Oncology Rostov‐on‐Done Russia; ^18^ Kazakh Institute of Oncology and Radiology Almaty Kazakhstan

**Keywords:** metastatic renal cell carcinoma, overall survival, registry, treatment patterns

## Abstract

**Background:**

Real‐world data describing outcomes of treatment among metastatic renal cell carcinoma (mRCC) patients are limited and heterogeneous.

**Aim:**

RENSUR3 registry study assessed real‐world data on the use of therapies in mRCC and overall survival (OS) in Russia, Kazakhstan, and Belarus.

**Methods:**

Patients were included in the retrospective multicenter registry study. To be eligible, patients were required to have mRCC diagnosed from January 2015 to January 2016. Anonymized data were collected through an online registry. The outcomes of interest were patient characteristics, treatment patterns, and OS.

**Results:**

1094 mRCC patients were identified. Mean age was 62.3 (SD, 11.2) years. Four hundred and forty‐four (41%) patients were 65 years and older. Primary tumor has not been removed in 503 (46%) patients. Subtype of RCC based on WHO classification (clear‐cell or other) *has been* reported in 402 (37%) patients. In total, 595 (54.4%) patients received systemic therapy for metastatic disease. 58% of elderly patients (≥65) were not treated compared to 37% of younger patients. Cytokines and targeted therapy were used in 298 (50.1%) and 297 (49.9%) of 595 treated patients, respectively. Median OS was 11.9 months (95% CI 10.9‐12.9). The 1‐ and 3‐year OS rates were 49.6% and 19.3%.

**Conclusions:**

Half of patients received no systemic therapy or had only cytokines for mRCC in Russia, Kazakhstan, and Belarus, which doubtless negatively affected OS in this population. Novel therapies should be considered as life prolonging and a priority.

## 1 INTRODUCTION

Real‐world data describing outcomes of treatment among metastatic renal cell carcinoma (RCC) patients is limited and heterogeneous. Russia, Kazakhstan, and Belarus are countries of the Eurasian Economic Union with upper‐middle‐income economies. In 2015, the largest absolute number of new cases of kidney cancer was diagnosed in Russia (22 846) comparing with Kazakhstan (1104) and Belarus (2261).^1^, ^2^, ^3^, ^4^ The European age‐standardized incidence rates per 100 000 population were 9.77, 6.05, and 14.8, respectively. To date, there is no exact information on how many patients have metastatic kidney cancer. A total of 5302 kidney cancer deaths were recorded in Russia, 373 in Kazakhstan, and 596 in Belarus.

RENSUR3 registry study assessed real‐world data on overall survival (OS) and the use of different treatment approaches in patients with newly diagnosed metastatic RCC.

## 2 METHODS

### 2.1 Patients

Patients from Russia, Kazakhstan, and Belarus were identified in the retrospective multicenter registry study. Centers are located in north, south, central and east parts of the Russia, all regions of Kazakhstan, and single comprehensive N.N. Alexandrov National Cancer Centre of Belarus. Anonymized data were gathered by oncologists through an online registry covering demographics, treatments, and outcomes. Patients were included if metastatic RCC was diagnosed from January 2015 to January 2016. To be eligible, patients were required to meet the following inclusion criteria: histologically proven metastatic RCC and aged ≥18 years at the time of diagnosis. Biopsy of primary tumor was performed before systemic therapy in patients without nephrectomy to confirm RCC. Patients treated as part of clinical studies were not eligible.

All procedures performed in RENSUR3 study involving human participants were in accordance with the ethical standards of the institutional and/or national research committee and with the 1964 Helsinki declaration and its later amendments or comparable ethical standards.

### 2.2 Outcome variables

The primary end‐point of study was evaluation of 3‐year OS. The other outcomes of interest included median OS, patient characteristics, and treatment patterns (surgical and systemic approaches used).

Progression of disease was evaluated with clinical and radiological investigation as well as markers of progression defined as therapy change and death. Patterns of switching from the first‐ to subsequent lines of systemic therapy were also assessed. Switch to subsequent treatment was defined as a switch due to disease progression or safety concerns. Some patient records did not include data for all parameters thus available data from these patients were used.

### 2.3 Statistical analysis

This is a retrospective cohort study. Summary statistics (mean, median, and proportion) were used to describe baseline patient characteristics and treatment patterns. Survival times were calculated from the date of therapy initiation to the date of death (OS). Survival curves were estimated using the Kaplan‐Meier method. Relationships between outcomes, demographic factors, and treatment patterns were assessed using Kaplan‐Meier analyses and log‐rank comparisons. All statistical analyses were carried out using IBM SPSS Statistics Base v22.0 (SPSS, Inc., Chicago, IL).

## 3 RESULTS

### 3.1 Patient characteristics

Overall, 1094 adult RCC patients were included for analysis. There were no excluded patients in this study. The mean number of patients in Russia, Kazakhstan, and Belarus was 573, 250, and 272, respectively. All patients had metastatic disease. Mean age at diagnosis of metastatic RCC was 62.3 (SD, 11.2) years. Four hundred forty‐four (41%) patients were 65 years old and older. More than half of the patients were male. Primary tumor has not been removed in 503 (46%) patients. Subtype of RCC using the World Health Organization classification (clear cell or other) *has been* reported in 402 (37%) patients. Data on MSKCC risk group were available in 447 (41%) patients. The patient characteristics are summarized in Table 1.

**TABLE 1:** Patient characteristics

	Russia	Kazakhstan	Belarus
	*N* = 573	*N* = 250	*N* = 272
**Age (range), years**	63.1 (24‐95)	60.8 (21‐79)	62 (18‐88)
<65 years old, *N* (%)	315 (55)	159 (64)	174 (64)
>75 years old, *N* (%)	258 (45)	91 (36)	98 (36)
**Sex, *N* (%)**			
Male	376 (65.6)	178 (71.2)	194 (71.3)
Female	197 (34.4)	72 (28.8)	78 (28.7)
**Previous surgery, *N* (%)**			
Nephrectomy	413 (72)	81 (32.4)	174 (64)
**Subtype of RCC, *N* (%)**			
***Verified***	311 (54)	22 (8.8)	69 (25.4)
Clear‐cell RCC	262 (84)^1^	—	—
Non‐clear cell RCC	49 (16)^1^	—	—
***Not verified***	262 (46)	228 (91.2)	203 (74.6)
**MSKCC risk group, *N* (%)**			
Favorable	79 (26)^2^	18 (12)^2^	—
Intermediate	167 (56)^2^	42 (28)^2^	—
Poor	53 (18)^2^	88 (60)^2^	—
NA	274 (48)	102 (41)	272 (100)

Abbreviations: MSKCC, Memorial Sloan‐Kettering Cancer Center; NA, not available.

### 3.2 Treatment approaches

In total, 595 (54.4%) patients received systemic therapy for metastatic disease. Cytokines and targeted therapy were used in 298 (50.1%) and 297 (49.9%) of 595 treated patients, respectively. Fifty‐eight percent of elderly patients (≥65 years old) were not treated compared to 37% of younger patients. Cytokines were the most commonly used treatment in elderly patients (115 of 447 patients, 61%) while targeted therapy was more widely used in younger patients (223 of 648, 55%). There were no significant differences in the frequency of systemic therapy depending on gender (*P* > .1). Of those patients who received systemic treatment, only first‐line therapy was administered to 425 (71%) patients; 170 (29%) patients received two or more lines.

The main reason (79%) for treatment discontinuation was disease progression. Treatment discontinuations due to adverse events occurred in 121 patients (18%). There were no treatment‐related deaths in the study. Table 2 describes treatment approaches in each country.

**TABLE 2:** Systemic therapy for metastatic RCC

	Russia	Kazakhstan	Belarus
	*N* = 573	*N* = 250	*N* = 272
**Systemic therapy, *N* (%)**	307 (54)	136 (54.4)	152 (55.9)
Targeted therapy, *N* (%)	159 (27.8)	115 (46)	22 (8)
Cytokines (IFN), *N* (%)	147 (26)	0 (0)	130 (48)
Other therapy, *N* (%)	1 (0.2)	11 (4.4)	—
**Number of lines, *N* (%)**			
One	376 (65.6)	87 (75.7)	118 (87.5)
Two and more	197 (34.4)	28 (24.3)	34 (12.5)
**Systemic therapy in patients**			
**≥65 years old, *N* (%)**			
Yes	114 (44)	34 (38)	46 (43)
No	144 (56)	56 (62)	62 (57)
**<65 years old, *N* (%)**			
Yes	192 (61)	102 (64)	106 (65)
No	123 (39)	58 (36)	58 (35)

Abbreviation: IFN, interferon.

At the time of the study, there were no clinical studies on RCC in these regions.

### 3.3 Overall survival

**FIGURE 1:**
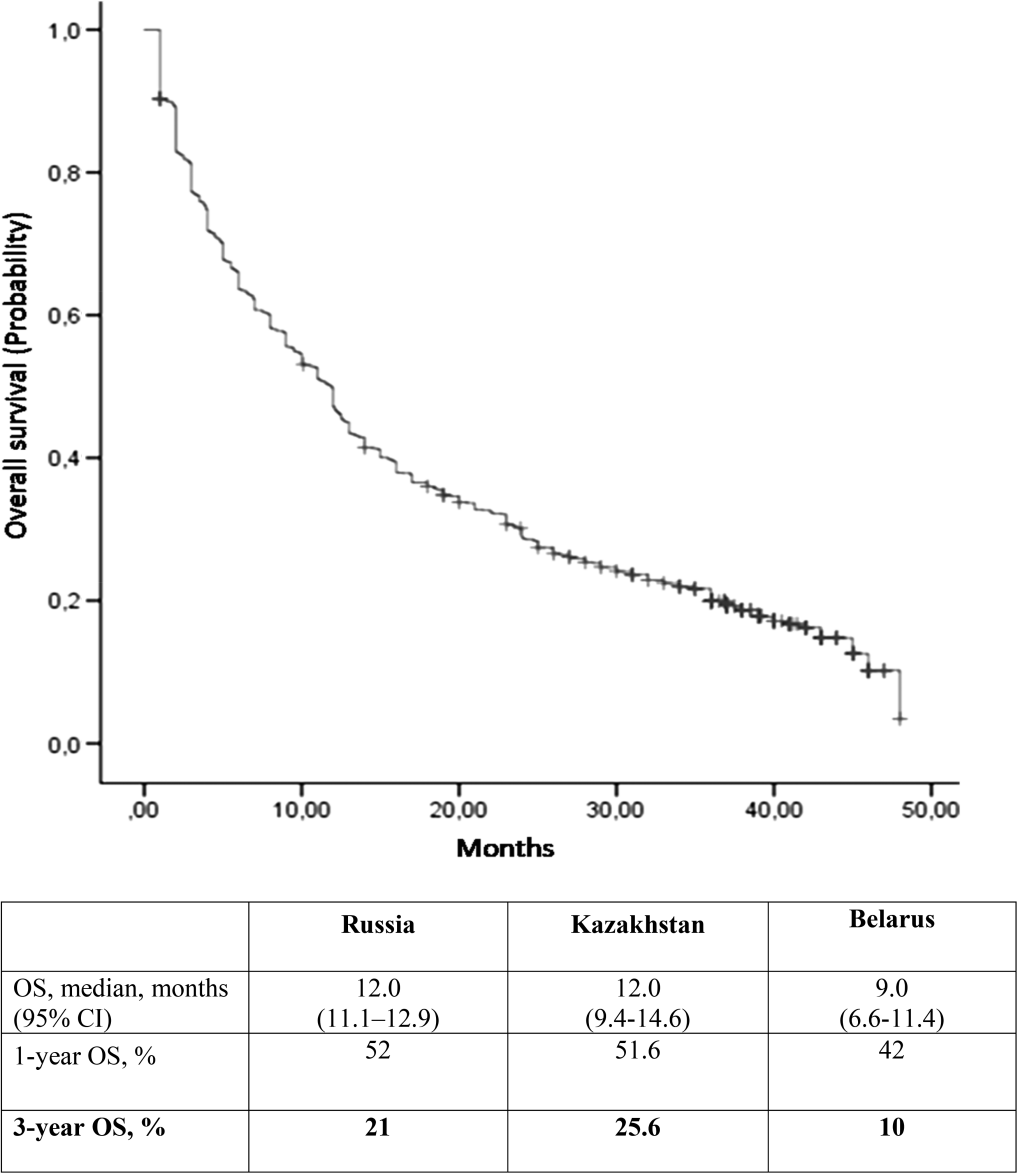
Kaplan‐Meier survival plot of OS in RENSUR3 registry study. Overall survival of patients with metastatic RCC in Russia, Kazakhstan, and Belarus

The 3‐year OS rate was 19.3%, with a median follow‐up period of 42 months. The 1‐year OS rate was 49.4%. The median OS from the start of treatment was 11.9 months (95% CI 10.9‐12.9). Survival curve is shown in Figure 1.

In assessing the relationship between survival, treatment patterns, and individual demographic characteristics, the survival of patients who received the second‐line of therapy (median 17.2 months) was significantly longer than patients who received only the first‐line of therapy (9.4 months, *P* < .001). Of the 184 patients who were alive at the last follow‐up, 120 (65.2%) patients received 2 and more lines of targeted therapy. The median OS was 12.7 months (95% CI 11.3‐14.1) and 9.3 months (7.7‐9.9) in patients aged <65 and ≥65 years, respectively (*P* < .0001). There were no significant gender differences in OS (*P* > .05).

## 4 DISCUSSION

In the RENSUR3 study, the survival of patients with metastatic RCC and factors affecting OS were estimated in eight representative regions of Russia, all regions of Kazakhstan, and comprehensive national cancer center of Belarus. Despite the fact that the exact number of patients with metastatic kidney cancer in each country is not known exactly, the analyzed population was representative and included an analysis of more than 10% of the regions. In total, data from more than a thousand of patients were collected and analyzed. The median OS calculated by the Kaplan‐Meier method in the entire population of the RENSUR3 study was 11.9 months, and 3‐year survival rate was 19.3%. Survival results were very similar between the three countries. These outcomes are comparable with the register data from other developing countries, but inferior to developed ones. For example, in the BRICS countries, the median OS of patients with metastatic RCC was 12.87 (India^5^) and 14.1 (Brazil^6^) months. Analysis of the multicenter Korean registry showed a median OS of 31 months in patients with clear cell metastatic RCC and 24 months in patients with non‐clear cell RCC.^7^ In the Czech Republic, the median OS was at least 26.3 months according to the data of the systemic therapy register (N = 1315).^8^ According to the results of the SEER 182010‐2016 register, 5‐year OS rate of patients with metastatic RCC was 13% in the United States.^9^ In 2016, Canadian patients treated with sunitinib or pazopanib had median OS of 21‐32 months.^10^ The total median cost of first‐ and second‐line treatments was around $50 000. Unfortunately, this cost of treatment could have been significant for developing countries at the time, and not all patients could be provided with targeted therapy. Interestingly, the gradual implementation of new therapies into daily practice has also improved survival rates over the years in North America. OS was significantly longer in the late than in the early targeted therapy era.^11^ Race differences can also affect survival. From a total of 3533 deaths among patients with clear‐cell metastatic RCC, 2684 occurred in the Caucasian population, 580 in the Hispanic population and 269 in the African American population in the United States.^12^ However, African American patients with clear‐cell metastatic RCC had a lower median OS compared with Caucasian patients and also had a higher overall mortality risk than Caucasian patients. Given that the countries in our study are populated by people of different nationalities, it is important to consider the fact of race in future research.

It should be noted that targeted therapy is prescribed regardless of age in North America that differs from approaches in Russia, Kazakhstan, and Belarus, where patients over 65 years of age had a lower chance of receiving systemic therapy, which apparently led to a decrease of survival in this group. The positive influence of targeted therapy on the survival of elderly RCC patients have also been shown in US retrospective cohort study.^13^, ^14^

Modest survival rates in the RENSUR3 study also explained by the use of systemic therapy only in half of the patients (54.5%), low frequency of targeted agents prescription (27%), limited administration of the second and subsequent lines in patients with the disease progression (29%), and lack approval of novel therapies (nivolumab, nivolumab/ipilimumab, pembrolizumab / axitinib, cabozantinib, lenvatinib/everolimus) at that time. The insufficient reimbursement could affect the access of RCC patients to treatment in 2015. The same problems are noted in other BRICS countries, in which only 20% of patients received second‐line therapy.^5^, ^15^ It is obvious that the subsequent lines of therapy are extremely important in treatment algorithm of metastatic RCC. In the German prospective registry study STAR‐TOR, median OS was 38.1 months in the patients receiving sequential therapy with sunitinib/temsirolimus and then axitinib, and 13.7 months in patients treated with first‐line therapy only (P < .0001).^16^ In the Czech RENIS register, the use of two and three lines of therapy resulted in a median survival of 29 and 50.9 months, respectively.^17^ During follow‐up period of 5 years, the median OS was 17.4 months in patients receiving second‐line everolimus in the Russian study CRAD001LRU02T, and the 3‐year survival rate was 43%.^18^

The treatment of metastatic RCC has been changed in 2015 with new treatment options significantly improving outcomes. The approval of novel targeted and immunotherapies after RENSUR3 study should be reflected in the changing patterns of daily treatment approaches. According to a long‐term observation from phases 1 and 2 clinical trials, approximately one third of patients with metastatic RCC who received nivolumab as part of the second and subsequent‐line therapy were alive after 4 or 5 years.^19^ Three‐year OS rate was 35%. A prospective study NIVOREN GETUG‐AFU 26 showed a median OS of 24.5 months and 1‐year survival rate of 69% in 729 patients treated with nivolumab in real‐world practice (patients had 2 and more previous lines of therapy, brain metastases, poor prognosis, impaired renal function, and so on).^20^ Similar figures are cited by the authors of the expanded‐access study, in which the 1‐year OS was 63%.^21^ The results of nivolumab in real‐world setting were consistent with the data from the CheckMate 025 pivotal study that demonstrated a median OS of 25.8 months and the 3‐year OS of 39%.^22^, ^23^ The revolutionary achievements of the first‐line therapy should be used in routine practice. A combination of nivolumab and ipilimumab resulted in 3.5‐year OS rate of 52% in intermediate and poor‐risk patients^24^ as well as the use of pembrolizumab and axitinib combination resulted in 74% of 2‐year OS in all risk patient group.^25^

It is worth noting that over the years, the system of reimbursement of novel medicines for the treatment of RCC has improved in each of the three countries. For example, in Russia, all treatment options that are recommended by European Society for Medical Oncology have been approved and become available. In Russia, Kazakhstan, and Belarus, medicines are provided at the expense of the state. However, in order to draw conclusions about how much the situation has improved; a new analysis with OS assessment will be required several years later.

A limitation of this study is in its retrospective design, as well as in a lack of complete data on IMDC risk group and inability to carry out multivariate analysis. However, the main goal was to assess survival in the general population of all mRCC patients who appeared in a given region. We want to emphasize that each center included all patients in the region, that is, we know the real OS rate in this population, which is important for these countries. Real‐world data could help improve regulatory decisions.

In conclusion, the results of the RENSUR3 study indicate the need for the further implementation of modern approaches in real‐world practice in order to significantly improve the short‐ and long‐term survival of patients with metastatic RCC in Russia, Kazakhstan, and Belarus. Most patients should receive systemic therapy regardless of age. In addition, access to second‐line and subsequent therapy should be provided.

## CONFLICT OF INTEREST

Authors declare that they have no conflict of interest.

## AUTHOR CONTRIBUTIONS

**Ilya Tsimafeyeu:** Conceptualization; data curation; formal analysis; funding acquisition; investigation; methodology; project administration; resources; software; supervision; validation; writing‐original draft; writing‐review and editing. **Oxana Shatkovskaya:** Conceptualization; data curation; formal analysis; investigation; methodology; project administration; validation; writing‐original draft; writing‐review and editing. **Sergei Krasny:** Conceptualization; data curation; formal analysis; investigation; methodology; project administration; resources; software; supervision; validation; writing‐original draft; writing‐review and editing. **Nurzhan Nurgaliev:** Data curation; formal analysis; investigation; writing‐review and editing. **Ilya Varlamov:** Data curation; formal analysis; investigation; writing‐review and editing. **Vladislav Petkau:** Data curation; investigation; writing‐review and editing. **Sufia Safina:** Data curation; investigation; writing‐review and editing. **Ruslan Zukov:** Data curation; investigation; methodology; writing‐review and editing. **Mikhail Mazhbich:** Investigation; writing‐review and editing. **Galina Statsenko:** Data curation; investigation; writing‐review and editing. **Sergey Varlamov:** Data curation; investigation; writing‐review and editing. **Olga Novikova:** Data curation; investigation; writing‐review and editing. **Igor Zaitsev:** Investigation; writing‐review and editing. **Pavel Moiseyev:** Investigation; writing‐review and editing. **Alexander Rolevich:** Data curation; formal analysis; investigation; writing‐original draft; writing‐review and editing. **Alesya Evmenenko:** Data curation; formal analysis; investigation; writing‐review and editing. **Irina Popova:** Investigation; writing‐review and editing. **Dilyara Kaidarova:** Conceptualization; data curation; investigation; project administration; writing‐review and editing. **Liubov Vladimirova:** Conceptualization; data curation; formal analysis; investigation; project administration; validation; writing‐original draft; writing‐review and editing.

## ETHICAL STATEMENT

The study protocol was approved by the principal investigators and ethics committee at each center. The patients provided informed consent.

## Data Availability

Data sharing is not applicable to this article according to local law.
